# The Pandemic and Older Women in the United States: Impacts on Social Networks and Well-Being

**DOI:** 10.1093/geroni/igab046.1972

**Published:** 2021-12-17

**Authors:** Lindsay Ryan

**Affiliations:** University of Michigan, Ann Arbor, Michigan, United States

## Abstract

The current study examines the unique impacts of the ongoing COVID-19 Pandemic on the well-being of middle aged to older women from the 2020 Health and Retirement Study (n = 1252) and how their reports of social contact during the pandemic compare to age-matched women from 2018 (n = 2063). Although up to a third of women across age categories reported changes in social contact due to the pandemic, their rates of communication with friends and family were not significantly different from their counterparts in 2018. Results find expected age patterns in satisfaction with life during the pandemic), where the young-old report the highest levels. However, the association of life satisfaction with the extent to which women reported more loneliness during the pandemic was only significant among the young old and oldest old. Age differences in pandemic-specific experiences in relation to well-being are discussed within a life course developmental framework.

